# Converting Energy with Glycerol and CO_2_ in a Microfluidic Fuel Cell Equipped with CuBiO_4_/CuO Photocathode: Bypassing Bubbles Challenge of Concurrent Water Splitting

**DOI:** 10.1021/acsomega.4c05943

**Published:** 2024-10-15

**Authors:** Silvio M. Mazarin, Daniel F. Costa-Filho, Cinthia R. Zanata, Adailton C. Nogueira, Maria-Victória S. Silva, Heberton Wender, Cauê A. Martins

**Affiliations:** Institute of Physics, Universidade Federal de Mato Grosso do Sul, CP 549, 79070-900, Campo Grande, MS, Brazil

## Abstract

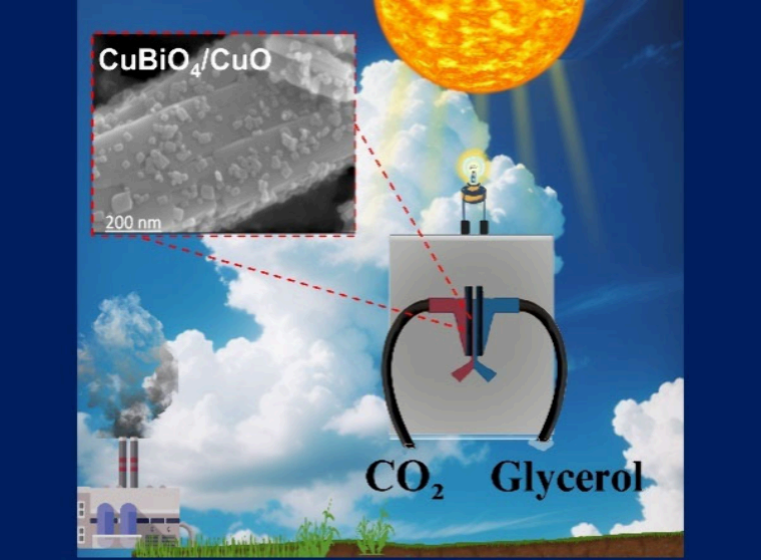

The imperative to address CO_2_ emissions has prompted the search for alternative approaches to capture this gas with minimal energy consumption. In this context, leveraging the CO_2_ reduction reaction (CO_2_RR) as an oxidant in fuel cells has emerged as a sophisticated strategy to convert this gas into usable energy. This study introduces a hybrid microfluidic photo fuel cell (μPFC) designed for the efficient conversion of CO_2_ and glycerol into electrical energy. The prototype integrates 3D-printed components with glass sealing, enabling precise control over the reactant flow and the use of light-sensitive catalysts. The anodic glycerol electrooxidation was investigated on Pt/C dispersed on carbon paper (CP), while the CO_2_RR was carried out on CuBiO_4_/CP and CuBiO_4_/CuO/CP in the presence of solar light. Half-cell measurements demonstrate the photoactivity of CuBiO_4_/CuO/CP and CuBiO_4_/CP electrodes for the CO_2_RR under light exposure at low onset potential in a neutral pH solution, generating a positive theoretical open-circuit voltage of 0.89–0.91 V when coupled to glycerol electrooxidation in an alkaline medium. The use of the mixed medium in the membraneless system equipped with the photosensitive catalysts allowed the building of this galvanic cell. However, the feasibility of using CuBiO_4_/CP is hindered by the disruption of the colaminar channel caused by hydrogen bubbles produced during concurrent water splitting. In contrast, the μPFC equipped with a CuBiO_4_/CuO/CP photocathode demonstrates a stable and reproducible performance, delivering a maximum power density of 0.9 mW cm^–2^. The formation of the CuBiO_4_/CuO heterojunction effectively suppresses photocatalytic water splitting, allowing for efficient CO_2_ conversion without disruption of the laminar flow channel. This innovative approach highlights the potential of μPFCs as sustainable energy converters for the utilization of CO_2_ in aqueous solutions, offering a pathway toward carbon-neutral energy production.

## 1 Introduction

The use of fossil fuels leads to the emission of large amounts of greenhouse gases into the atmosphere, causing a constant accumulation of CO_2_ and, consequently, an imbalance in the carbon cycle. In 2024, the Earth’s surface and ocean experienced a global average temperature of 1.27 °C above the 20th-century average of 12.2 °C, marking it as the warmest January on record in the 175-year global climate history. This surpassed the previous record set in January 2016 by 0.04 °C.^1^ The IPCC (Intergovernmental Panel on Climate Change) suggests that reaching the peak of global greenhouse gas emissions by 2025 at the latest and subsequently reducing them by 43% by 2030 represent the most effective strategy for limiting warming to 1.5 °C.^2^ Hence, it is evident that there is a pressing need to mitigate carbon emissions by both preventing their release and capturing of CO_2_.

In this context, electrochemical technologies emerge as potential devices for transforming CO_2_ into other less-harmful organics that are potentially useful in industrial applications.^3,4^ For example, recently Peña-Rodriguez et al.^5^ investigated the production of formate from CO_2_ in the cathode coupled to the glycerol electrooxidation in the anode using an unprecedented reactor with a single-pass arrangement operating in continuous mode. The HCOO^–^ production at the Bi gas diffusion cathode reached a Faradaic Efficiency of 93% with low power consumption (208 kW h kmol^–1^), while 283 μmol m^–2^ s^–1^ of dihydroxyacetone was produced from glycerol on Pt anode modified with Bi_2_O_3_.^5^ Kalaramas et al.^6^ converted CO_2_ into methanol and formate in a continuous flow photoelectrochemical microfluidic reactor. The authors used CuO-based thin films as photocathodes under simulated AM 1.5 solar irradiation for up to 12 h. The highest photocurrent density obtained was for the α-Fe_2_O_3_/CuO photoelectrode yielding −1.0 mA cm^–2^ at 0.3 V vs RHE, and initial results indicated a solar-to-fuel efficiency of 0.48%. The CuO, Cu_2_O, and CuO–Cu_2_O photoelectrodes were selective for formate generation, while the bilayered α-Fe_2_O_3_/CuO photocathode produced formate and methanol.^6^ Recent studies have demonstrated a membraneless flow microfluidic electrolyzer operation equipped with a Ag cathode and Pt/C anode for converting CO_2_ coupled with a glycerol electrooxidation reaction. By replacing the oxygen evolution reaction (OER), the normal anode process applied in CO_2_ electrolyzer cells, the required cell voltage decreased by almost 1 V, operating at ca. 200 mA cm^–2^ current density with stable selectivity for 5 h.^7^

These devices work by applying a potential to drive the CO_2_ reduction reaction (CO_2_RR) at the cathode, while a reducing agent undergoes oxidation at the anode, within an electrolysis process. Consequently, a bias is applied to promote a nonspontaneous reaction. Another optimal process involves the spontaneous operation of the CO_2_RR at the cathode, driven by the oxidation of fuel at the anode within a fuel cell configuration. In this approach, the coupled reactions occur spontaneously at zero bias, enabling the conversion of aqueous CO_2_. The main challenge to converting CO_2_ in a fuel cell lies in the high onset potential (*E*_onset_) required for the CO_2_RR, which prevents its practical application. In this context, microfluidic fuel cells (μFCs) emerge as a powerful solution.

A μFC is a miniaturized fuel cell engineered with the same work principles, containing all parts within a microfluidic channel.^8,9^ Unlike traditional fuel cells, this design offers advantages such as the use of a laminar flow to separate the anode from the cathode, eliminating the need for a membrane and the associated ohmic loss. This design also permits the use of anolyte and catholyte at varying pH levels,^10^ enabling the selection of the most optimal individual half-cell configuration. Furthermore, the flow configuration^11^ constructed with porous electrodes enhances surface utilization, mass transport, and electrochemical conversion.^12^ Additionally, μFCs have the flexibility to utilize a variety of catalysts, including metallics or semiconductors.

As semiconductor materials are cheaper than noble metals, the substitution of one of the dark electrodes (anode or cathode) with a semiconductor material has attracted the interest of researchers.^12−15^ In this scenario, the development of microfluidic photocatalytic fuel cells (μPFCs) has emerged. μPFCs result from the integration of photocatalysis and μFCs. They are miniaturized systems that convert chemicals into electrical energy under light irradiation, using the same concept and advantages of microfluidic systems.^15^

Hence, equipping μPFCs with a photocathode enables CO_2_ reduction, while fuel oxidation occurs at the anode, yielding power. Adjusting the pH levels for each half-cell reaction facilitates a positive cell voltage and subsequent negative Gibbs free energy. However, a prevalent challenge arises as many catalysts efficient for CO_2_RR also catalyze the hydrogen evolution reaction (HER) in aqueous solutions.^3,16^ The adsorbed hydrogen binding affinity closely correlates with that of carbon-binding intermediates, underscoring the importance of selectively suppressing HER over CO_2_RR. This becomes particularly crucial in scenarios where the catalyst-electrolyte interface exhibits a high surface.^3^

Gaseous species like H_2_ lead to bubble formation on the electrode surface, causing significant energy losses by obstructing ionic conduction paths, reducing the electrocatalytic area, and disturbing concentration gradients at the electrode–electrolyte interface. Moreover, the presence of bubbles disrupts the colaminar flow, causing disturbances in the ionic transfer at the interface of the two flows. Angulo et al. used a microfluidic water electrolyzer to examine how electrochemical reaction conditions and convective flows affect bubble-induced overpotential losses.^17^ The researchers demonstrated that laminar flow with a higher Reynolds number (Re > 20) helps reduce the formation of large bubbles, resulting in minimal overpotential losses. Laminar flows with lower Re numbers, on the other hand, showed periodic formation of large bubbles leading to fluctuations in overpotential of around 100 mV.^17^ Additionally, the authors used fluorescence microscopy and pH-sensitive dyes to analyze the impact of bubbles on concentration overpotentials, revealing that large bubbles at low Re numbers can cause more severe concentration gradients influenced by hydrodynamic flows.^17^

Here, we introduce a novel hybrid μPFC generation integrating 3D-printed components with glass sealing for efficient CO_2_ conversion. The cell features a semiconductor optimized for the cathodic reaction, tailored to minimize activity toward the HER, thereby ensuring operational stability. For that, we redesigned CuBi_2_O_4_ modified with CuO,^18^ for CO_2_RR, while electrons are harvested from glycerol electrooxidation at the anode. This pioneering approach enables the development of the first μPFC capable of converting CO_2_ into energy in aqueous environments.

## 2 Experimental Section

### 2.1 Synthesis and Characterization of CBO and CBO/CuO Heterojunction by Solvothermal Method

Inspired by the high activity of CuBi_2_O_4_/CuO heterojunctions toward pollutant model photodegradation,^18^ we designed these materials as photocathodes for a new application. From now on, the synthesized CuBi_2_O_4_ particles will be called CBO. The precursors were synthesized according to the methodology described by Nogueira et al.^18^ The CBO/CuO heterostructures were prepared in a single-step solvothermal reaction from the precursors bismuth nitrate pentahydrate and copper acetate monohydrate using ethanol and water as solvents. Figure S1 presents the synthesis route. In a typical synthesis CBO/CuO, 25 mM Bi(NO_3_)_3_·5H_2_O, and 18 mM of Cu(CH_3_COO)_2_·H_2_O were dissolved separately in 36 mL of water/ethanol (1:1 v/v), resulting in two solutions—one white and the other dark blue. These solutions were subsequently mixed and stirred magnetically for 20 min. Subsequently, 111 mM sodium hydroxide was added to the mixture mentioned, resulting in the formation of a dark green solution. The final solution was transferred to a Teflon-lined stainless-steel autoclave with a total 110 mL volumetric capacity and annealed at 120 °C for 12 h at a heating rate of 10 °C·min^–1^. After spontaneous cooling, the precipitate was collected, washed three times with deionized water and three times with ethanol, and then dried at 80 °C for 8 h. The procedure was appropriately modified for the synthesis of CBO, where bismuth nitrate was first dissolved in 10 mL of a 2 mol L^–1^ HNO_3_ solution before being mixed with the Cu acetate solution (12.5 mM) and NaOH (100 mM). Following the same procedure described above, CBO in pure phase was obtained and stored.^18^

Thus, CBO and CBO/CuO were physically and chemically investigated. The morphology was studied by Field-Emission-Gun Scanning Electron Microscopy (FESEM) using Zeiss Sigma equipment. The powder samples were dispersed on the surface of carbon tape and mounted on the top of a standard aluminum alloy stub. FESEM images were acquired using an aperture size of 60 μm, an acceleration voltage of 15 kV, and a working distance of ∼8 mm. The crystallography was investigated by X-ray diffractometry (XRD) in a Bruker D2 Phaser diffractometer, configured for Cu Kα radiation (λ = 1.54 Å) in the angular range of 20 ≤ 2θ ≤ 80°, with angular steps of 0.02° and counting 5 s at each point, while the surface chemical composition was studied by X-ray photoelectron spectroscopy (XPS) in a conventional XPS instrument (Scienta Omicron ESCA+) with a high-performance hemispheric analyzer (EAC2000) with monochromatic Al Kα (*hν* = 1486.6 eV) radiation as the excitation source. The operating pressure in the ultrahigh-vacuum chamber (UHV) during the analysis was 1 × 10^–9^ Pa. The XPS high-resolution spectra were recorded at a constant pass energy of 50 eV with 0.05 eV per step. The data were processed using the *CasaXPS* software (Casa Software Ltd.). A Shirley background subtraction was performed before the curve fitting for all the data, and the peak positions were corrected by C 1s adventitious carbon set at 284.8 eV. Valence band XPS data were measured in high-resolution mode and calibrated by linear extrapolation of the signal to zero intensity. Charging effects were eliminated by using a low-energy electron flood gun.

### 2.2 Half-Cell Electrochemical Experiments

The photosensitive materials are first dispersed on carbon paper (CP) Toray 060 for electrochemical applications. The porous electrodes were prepared by cutting 15 × 6 mm CP for half-cell and cell measurements. These thermally treated strips were immersed in a catalytic ink dispersion (17.5 mg of catalyst + 672.37 μL of DI water + 672.37 μL of methanol + 155.25 μL of Nafion) in an ultrasonic bath for 5 min and dried at 80 °C for 4 h. All electrodes were similarly prepared using powder of CBO, CBO/CuO, or Pt/C (57% E-TEK). Thus, we investigated the black anode Pt/C/CP and the photosensitive cathodes CBO/CP and CBO/CuO/CP. The electrochemical measurements were performed in a polymeric three-electrode cell equipped with a quartz window (Figure S2). A high-area Pt plate was used as a counter electrode, and a reversible hydrogen electrode (RHE) prepared in the electrolyte (same solution) was a reference. The standard potential of the reference electrode was calculated using the Nernst equation *E* = *E°* + 0.0591 pH. The porous electrodes were used as a working electrode. All electrochemical measurements were performed in a Potentiostat/Galvanostat Interface5000 Gamry Instruments. The immersed part of the CP was 0.3 cm^2^, which was used for current normalization. The glycerol electrooxidation reaction (GEOR) was investigated using 1 mol L^–1^ glycerol + 1 mol L^–1^ O_2_-free KOH between −0.77 and 0.4 V vs SHE at 0.05 V s^–1^. The cathodic reactions were studied using a CO_2_-saturated + PBS pH 7 between 0.5 and −0.4 V vs SHE at 0.01 V s^–1^.

The system used for the half-cell measurements features a quartz window that allows light to interact with the photosensitive material (Figure S2). The top opening is sealed with rubber joints to facilitate atmospheric control during saturation with the working gases. Additionally, there are openings for the connection of the inlet and outlet for the working gases, as well as the installation of the working, reference, and auxiliary electrodes (Figure S2). All parts of the cell are screwable, allowing for easy disassembly and cleaning. The inlet openings facilitate quick connection of hoses and electrodes with glass casing, such as in the case of the hydrogen reference electrode and platinum auxiliary electrode (Figure S2). For the measurements in the presence of light, the photocatalyst was excited by using a solar simulator from ABET Tech with an AM1.5G filter and 150W Xe lamp, as shown in Figure S3. All measurements were calibrated with an ABET reference solar cell for an irradiance of 200 mW cm^–2^.

### 2.3 Additive Manufacturing and Sealing the Microfluidic Fuel Cell

We printed the plastic part of the cells using the Stereolithography Apparatus (SLA) technique, with a photosensitive resin type 3D cured in UV 404 nm in a printer Anycubic, model Photo Mono X. The cell was modeled in the software Autodesk Inventor 2023 and sliced in ChituBox. After printing, the pieces of the cell were conditioned in a washer Anycubic model Wash & Cure Plus for 10 min in isopropyl alcohol. Next, the pieces are cured in a rotational platform at UV 405 nm for 2 min. The platform allows the printing of 24 cells per batch. The total time for printing and post-treatment was 3 h and 45 min.

Unprecedentedly, we used glass compatible with photosensitive materials for sealing the μPFC. The microfluidic channel was placed on a glass typically used in optical microscopy, sealed onto the plastic piece with adhesive of touch screen protective film, with the aid of a Silhouette-type cutting machine—further detailed. The porous electrodes, prepared with different catalysts, were cut to have 1 mm × 10 mm dimensions and placed inside the microchannel. The cell was then closed with the glass plate adhered to the adhesive of the touch screens. Copper wires with Ag epoxy at their ends were used to connect to the electrodes. The inlet and outlet were connected using Tygon tubes. We used a double syringe pump (KDS Legato, model 101) to drive the anolyte and catholyte. The glass used to seal the μPFC was investigated by using a Shimadzu UV-2600i spectrophotometer. The spectrum was plotted in terms of transmittance.

### 2.4 Microfluidic Fuel Cell Tests

The μPFCs tests were performed with the assembly described in section 2.3, with the Pt/C/CP anode fed by an O_2_-free 1 mol L^–1^ glycerol + 1 mol L^–1^ KOH as the anolyte and the CBO/CP or CBO/CuO/CP cathode fed by CO_2_-saturated + PBS pH 7. All polarization measurements were performed with a potentiostat/Galvanostat Interface5000 Gamry Instruments, recording the current response from the OCV to 0.01 V at a scan rate of 0.01 V s^–1^ under 2 suns light exposure.

## 3 Results and Discussion

### 3.1 Physical-Chemical Characterization

**Figure 1:**
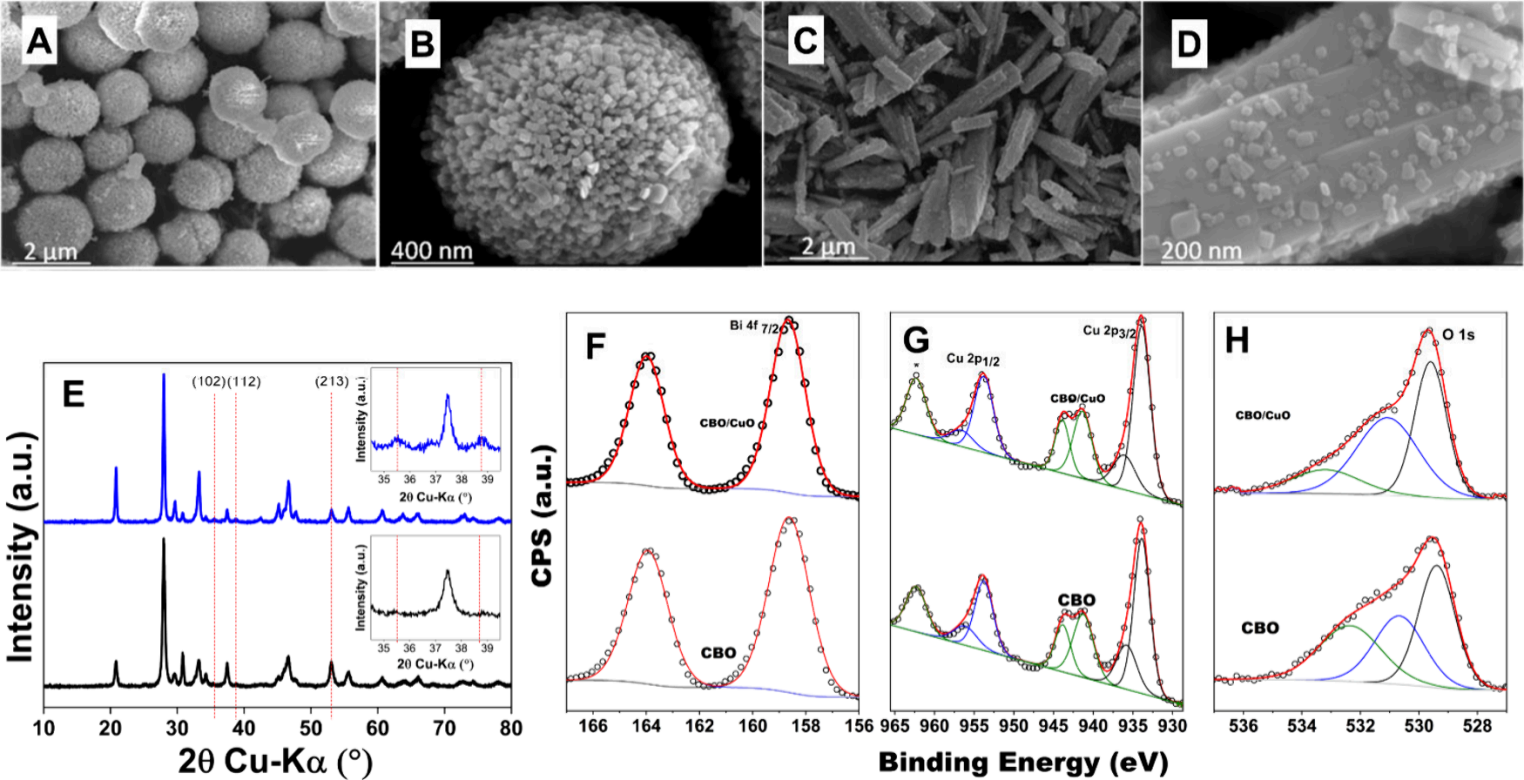
Catalyst characterization, featuring FESEM images of (a, b) CBO and (c, d) CBO/CuO. (e) XRD patterns of CBO/CuO (blue line) and CBO (black line). High-resolution XPS spectra of CBO and CBO/CuO heterojunction samples featuring (f) Bi 4f, (g) Cu 2p, and (h) O 1s regions. Data from the Figure taken with permission from J. Phys. Chem. C 2019, 123, 42, 25680–25690, copyright 2019.

Figure 1A–D shows FESEM images of the CBO and the CBO/CuO heterojunction. The CBO exhibits well-distributed microspheres formed by nanocolumn-shaped particles with an average diameter of 2 μm (Figure 1A,B), while the CBO/CuO sample consists of nanocolumns with an average length of 2 μm and diameter of 200 nm, with smaller platelike nanoparticles (around 60 nm) evenly dispersed on the surface (Figure 1C,D). The control of morphology appears to be influenced by the NaOH concentration during synthesis.^18^ As previously reported,^18^ EDS quantification results showed an expected Bi/Cu atomic ratio of approximately 2:1 for the CBO sample. For the CBO/CuO heterojunction, the ratio was around 2:2.2, indicating a higher amount of Cu in the heterojunction. Additionally, selected area EDS revealed that the ∼60 nm nanoparticles attached to the nanocolumn surface contained higher levels of copper, suggesting that these nanoparticles are likely composed of CuO.^18^

The phase formation of CBO and CBO/CuO was examined by XRD, as depicted in Figure 1E. From the pattern, the diffraction peaks match the CBO tetragonal phase (space group *P*4/*ncc*, PDF No. 72-493),^18^ clearly identified for CBO/CuO (blue line) and CBO (black line) in Figure 1E.^19^ For the semiconducting heterojunctions, XRD peaks confirm the presence of CuO through the peaks at 35.5°, 38.8°, and 53.0° corresponding to the (102), (112), and (213) CuO planes,^18^ respectively, shown in the inset in the blue line graph of Figure 1E, absent for CBO. The presence of CuO in the heterojunction was previously confirmed by Rietveld analysis, which identified 16.8 wt % of CuO and 83.2 wt % of CBO.^18^

The XPS analysis was conducted to explore the surface electronic structure and chemical state of CBO and CBO/CuO heterojunctions.^18^Figure 1F–H presents the high-resolution XPS spectra of Bi 4f, Cu 2p, and O 1s. The similarity of the Bi 4f XPS signals for CBO/CuO and CBO (Figure 1F) suggests that the excess Cu used to form the CBO/CuO heterojunctions does not alter the oxidation state of Bi. The two sharp photoelectron peaks centered at 158.7 and 163.9 eV are attributed to the binding energies of Bi 4f_7/2_ and Bi 4f_5/2_, of Bi^3+^ ions, respectively.^20−22^ The Cu 2p regions exhibit two shakeup satellites around 942 and 962 eV that confirm the presence of Cu^2+^.^23,24^ Additionally, two doublets were taken into consideration to accurately fit the Cu 2p_3/2_ and Cu 2p_1/2_ peaks (Figure 1G). These two contributions showed Cu 2p_3/2_ peaks centered at 933.9 and 935.8 eV (936.1 eV for CBO/CuO), respectively. The first peak is assigned to Cu^2+^ oxide, while the other peak is assigned to Cu^2+^ hydroxide [Cu(OH)_2_].^18,25^Figure 1H shows the O 1s spectra, which were decomposed into three peaks at 529.5, 531.1, and 532.5 eV for a pure sample of CBO. The main peak of O 1s at 529.5 eV is attributed to the contribution of oxygen in the lattice, while the others are related to surface defects, absorbed water molecules, or chemically absorbed oxygen species, as previously observed.^23,26^ Core-level energies of CBO and CBO/CuO were obtained from the XPS valence band spectrum and previously reported.^18^ Although presenting similar XPS features, the survey quantification results presented a higher Cu/Bi ratio for CBO/CuO surface composition compared to the CBO sample, corroborating with the formation of the Cu^2+^-based secondary phase in the heterojunction.^18^ After characterization, the catalysts were submitted to a half-cell reaction investigation and cell measurements.

### 3.2 Half-Cell Measurements

**Figure 2:**
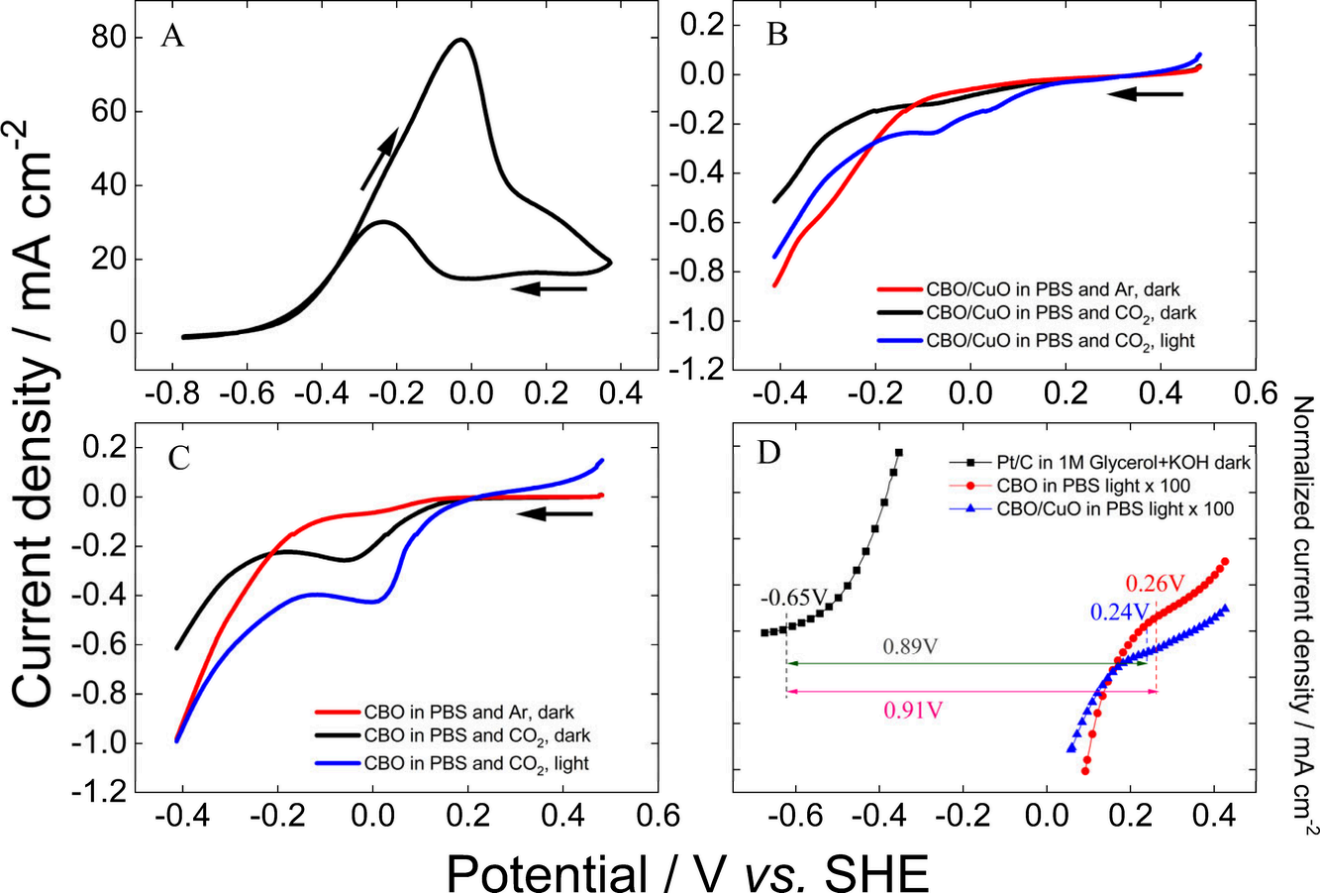
Electrochemical measurements in half-cell measurements. (a) Cyclic voltammogram of Pt/C/CP in the presence of 1 mol L^–1^ glycerol in 1 mol L^–1^ KOH at 0.05 V s^–1^. Linear voltammogram of (b) CBO/CuO/CP and (c) CBO/CP in PBS (pH 7) electrolyte saturated with Ar and in the presence and absence of CO_2_ saturated under dark and light conditions at 0.01 V s^–1^. (d) Details of the anodic and cathodic half-cell reactions highlighting the theoretical cell open-circuit potential, whether the reactions are coupled–cathodic currents multiplied by 100 to facilitate comparison. Measurements under light were performed at 200 mW cm^–2^ light intensity. The potentials of the reversible hydrogen electrode (RHE) prepared in the electrolyte (same solution) as a reference were corrected to the standard potential of the reference electrode, calculated using the Nernst equation *E* = *E*° + 0.0591 pH.

The GEOR was investigated on the Pt/C/CP electrode in an N_2_-saturated 1 mol L^–1^ glycerol in 0.1 mol L^–1^ KOH solution, as shown in Figure 2A. There are anodic currents on both positive and negative potential going scans.^27^ During the positive scan, electrooxidation starts at −0.65 V, reaching a maximum current density at −0.04 V. During the reverse scan, an oxidation peak appears at ca. −0.08 V, resulting from the surface reactivation after the reduction of surface oxides of Pt.

**Figure 3:**
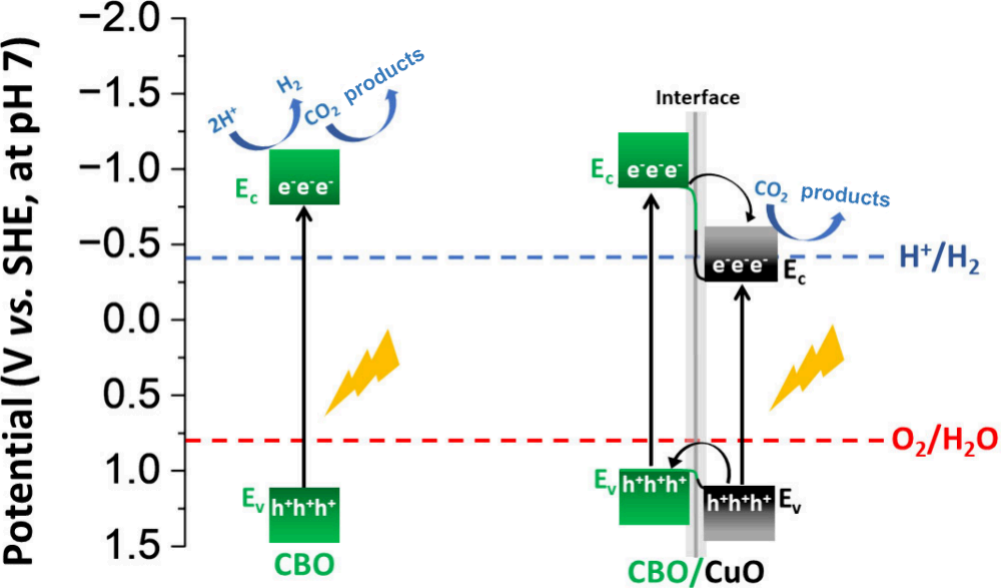
Energy band diagrams for CBO and CBO/CuO semiconducting heterojunction under visible light irradiation. The energy values used in the figure were determined in our previous publication.^18^ Data from the Figure taken with permission from J. Phys. Chem. C 2019, 123, 42, 25680–25690, copyright 2019.

The CO_2_RR was investigated in the absence and presence of light on the CBO/CuO/CP (Figure 2B) and CBO/CP (Figure 2C) electrodes. In the presence of light, both catalysts reveal photoactivity toward CO_2_RR starting at ∼0.24 V, while only CBO/CP shows some activity under dark (Figure 2C). These results reveal that CBO/CuO/CP and CBO/CP are active for the CO_2_RR in the presence of light. The photocurrent density has an obvious increase with applied potential (more negative values) reaching −0.24 and −0.40 mA cm^–2^ for CBO/CuO/CP and CBO/CP at −0.11 V, respectively, until a plateau at −0.21 V. After this point, the current densities abruptly increase even in the absence of light and CO_2_ due to the HER (Figure 2B,C), also reported in the literature.^28^ At −0.41 V, CBO/CP shows a current density of ∼0.98 mA cm^–2^ related to the HER, while CuO/CBO/CP delivers 0.73 mA cm^–2^. Even though both catalysts show similar current densities for the CO_2_RR, CBO/CP displays a higher current for water splitting. The reason for that lies in their electronic properties.^18^Figure 3 illustrates the band diagram before and after the formation of the heterojunction. Under solar irradiation, the electrons from the valence band (*E*_V_) of both CBO and CuO are excited to their respective conduction band (*E*_C_). However, due to the type-II energy band alignment in the CBO/CuO heterojunction, the electrons photoexcited in the *E*_C_ of CBO migrate to the conduction band of CuO, located at a more positive potential, and there it cannot induce the generation of H_2_ due to lack of the necessary energy to drive the 2H^+^/H_2_ reaction (−0.41 V vs SHE),^18^ since it is at a more positive potential. Thus, most of the HER found in CBO/CuO/CP comes from the HER on CP (Figure 2B). On the other hand, the excited electron in the *E*_C_ of pristine CBO has an energy more negative than the redox potential of 2H^+^/H_2_,^18^ facilitating HER (Figure 3). Furthermore, it is worth noticing that CBO/CuO/CP also displays HER to some extent since the reaction is not completely avoided, being part of the photoelectrocurrent shown for the CO_2_RR at 2 suns. However, most of the activity came from the CO_2_ reduction (Figure S4).

The onset potential of the reaction (*E*_onset_) is related to the energy required to initiate the reaction on the catalyst surface, making it an important electrocatalytic parameter. A galvanic cell is guaranteed by a negative free Gibbs energy variation (Δ*G* < 0), measured by Δ*G* = −*nFE*, where *n* is the number of moles of electrons, *F* is Faraday’s constant, and *E* is the cell potential calculated as *E*_onset_ at the cathode minus *E*_onset_ at the anode. Thus, *E* must be positive to allow a spontaneous overall reaction.

Through linear voltammograms, it was possible to determine *E*_onset_ as −0.65 V for the electrooxidation of glycerol and 0.26 and 0.24 V for the reduction of the CO_2_ reaction on CBO/CP and CBO/CuO/CP in the presence of light, respectively. Linear voltammograms for the anodic and cathodic reactions (Figure 2D) suggest theoretical open-circuit voltage (OCV) of 0.91 and 0.89 V when the anodic reaction is coupled with CO_2_ reduction on CBO/CP and CBO/CuO/CP, respectively. A positive OCV or *E* results in Δ*G* < 0. Consequently, these half-cell reactions can be coupled to produce energy spontaneously, with higher *E* indicating a more spontaneous reaction. This unprecedented result suggests that we can build an μPFC with these coupled reactions to produce power from glycerol and CO_2_.

### 3.3 Assembling the 3D-Printing Microfluidic Fuel Cell with a Light Transparent Glass

**Figure 4:**
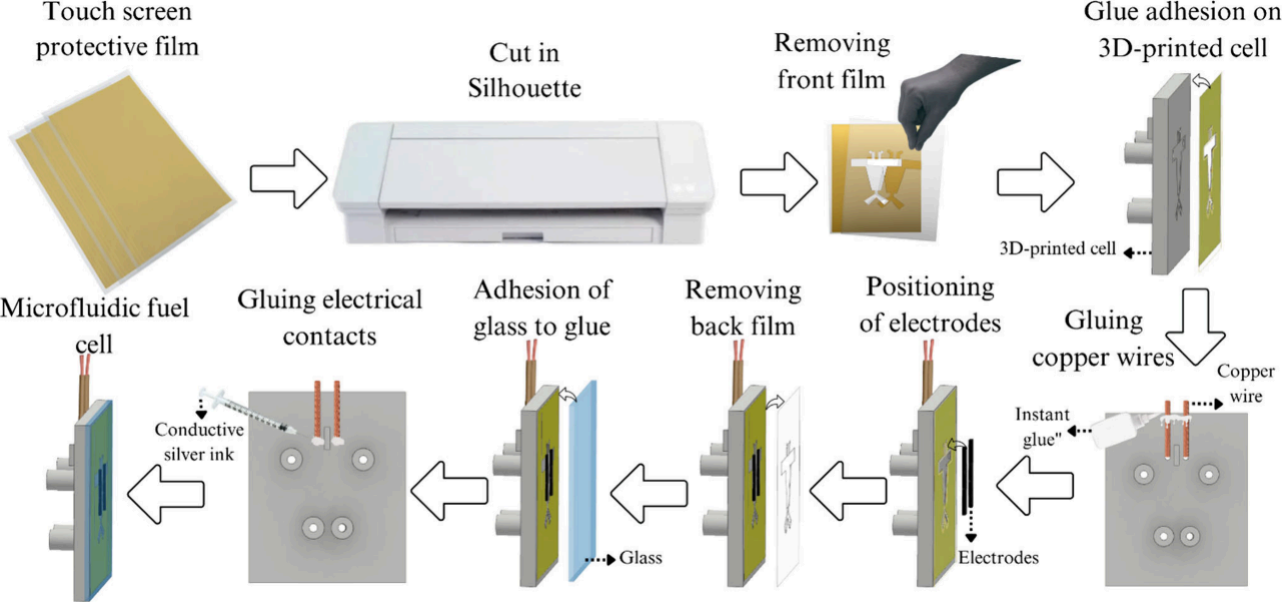
Details of the μPFC assembly, featuring all steps from cutting the touchscreen protective film to the placement of electrodes and sealing.

We present a new hybrid μPFC that combines 3D printing with the use of glass. Figure 4 details all of the steps for assembly. The strategy is to 3D-print the μFC model, specially designed to accommodate the porous electrolytes (Figure S5), place the electrodes, and cover the flat areas with an adhesive from a touch screen and a glass to seal it. The plastic front part of the cell was designed using Autodesk Inventor 2023 software, based on a pre-existing model developed by our research group.^29^

Seeing Figure 4, first, the touchscreen protective film is cut in a Silhouette following the microchannel model, yielding a three-layer cut piece, containing two plastic films sandwiching an inner layer of glue. Next, one front plastic film is removed exposing the adhesive layer, which is attached to the 3D-printed cell. At this point, Cu wires are fixed at the upper part of the 3D-printed cell, followed by placing the porous electrodes on the respective grooves (cathode and anode). The other outer plastic film of the touchscreen protective piece is removed making the bottom part highly adhesive; thus, the glass is attached, sealing the μPFC. Finally, the connection of the Cu wires and the porous electrodes is built using Ag epoxy and dried spontaneously for 4 h, building the μPFC.

The glass that seals the device and allows for the exposure of the photocathode to radiation had its transmittance spectrum analyzed. As shown in Figure S6, the transmittance within 400–700 nm is ∼91%, allowing for the absorption of photons by the photocatalyst across the entire visible light spectrum. Even though here we use CBO and CBO/CuO with excellent photoactivity within this wavelength range,^18^ the glass is 90% transparent to UV light between 360 and 400 nm (Figure S6), allowing one to use semiconductors active in the UV.

The stability of the colaminar channel was evaluated by flowing two dyes (2 drops of dye in 50 mL of water) at different flow rates, as shown in Figure S7. The glass used in sealing allows visual access to the colaminar channel. The laminar flow is stable in the range of 30–100 μL min^–1^, inducing constant interfacial flow throughout the length of the microchannel. The stability of the colaminar channel is crucial for maintaining constant resistance to ionic transport. To keep the cell working, it must have a low Reynolds’ number (Re), up to a few hundred, which is calculated as shown in eq 1:
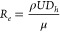
1where Re depends on the fluid density (ρ), average velocity (*U*), hydraulic diameter (*Dh*), and dynamic viscosity (μ). The μFC works at Re 0.36, 0.59, 0.89, and 1.14 for 30, 50, 75, and 100 μL min^–1^, respectively. The calculations for each flow are presented in Figure S8. This system was then used for the energy conversion tests.

### 3.4 Microfluidic Photo Fuel Cell Tests

Finally, we investigated the performance of the innovative μPFC assembled with a dark anode and photocathodes to convert energy from glycerol and CO_2_. The cell was fed with 1 mol L^–1^ glycerol + 1 mol L^–1^ KOH anolyte and pH 7 PBS solution of CO_2_-saturated catholyte at 30, 50, 75, and 100 μL min^–1^. We started the measurements with the μPFC equipped with a CBO/CP photocathode due to the well-defined and high current densities found in half-cell measurements (Figure 2C). Even though the system works, interestingly, it is hardly reproducible, as seen in Figure S9. Although it presented the best catalytic performance in half-cell experiments, the polarization and power density curves exhibit a significant amount of noise for all evaluated flow rates, which hinders the reproducibility and interpretation of the data. The reason for this is further rationalized.

**Figure 5:**
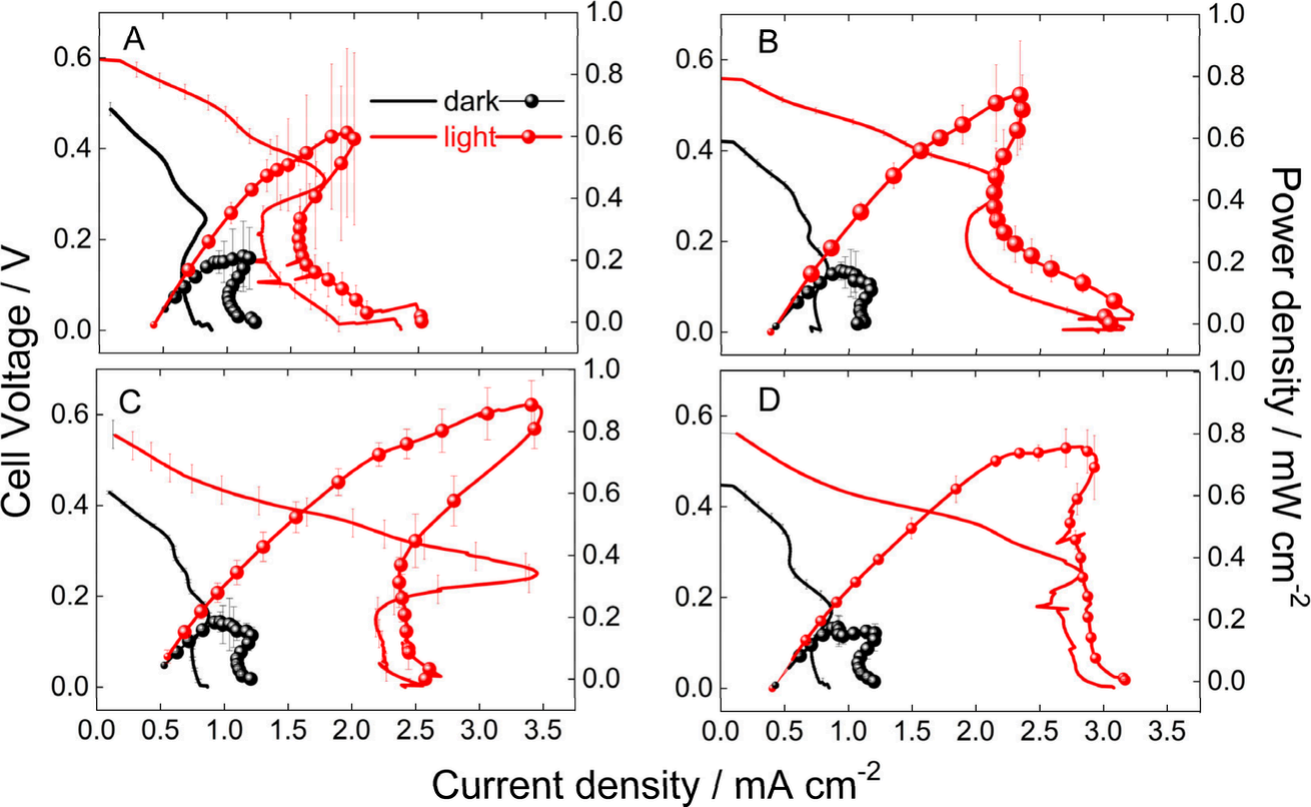
Polarization and power density curves of a μPFC equipped with a CBO/CuO/CP photocathode and Pt/C/CP dark anode fed by 1 mol L^–1^ glycerol + 1 mol L^–1^ KOH anolyte and CO_2_-saturated PBS catholyte at (a) 30, (b) 50, (c) 75, and (d) 100 μL min^–1^. Measurements were collected from the OCV to 0.01 at 0.01 mV s^–1^ under light induced by a solar simulator at 200 mW cm^–2^.

The performance of the device equipped with a CBO/CuO/CP photocathode displayed stable and reproducible polarization and power density curves, as shown in Figure 5. The use of a light-excited CBO/CuO/CP photocathode improves performance compared with the dark system in all studied flows (Figure 5A–D). The regimes of the polarization curve are well-defined. The activation polarization controls the cell voltage drop at low current densities, mostly due to the limited activity of Pt/C/CP for glycerol electrooxidation. The ohmic polarization controls most of the features in the whole range of current, up to a maximum, where the maximum power density is achieved, and the current density drops. This behavior can be ascribed to a change in the anodic pathway reactions, changing the number of electrons coming from the anode. The change in the reaction path can also be attributed to CO poisoning. A similar effect was previously observed for cells fed by formic acid^30,31^ and glycerol.^27,29^ When the current density stops dropping, the cell voltage suddenly drops due to starving at the cathode side; that is, the mass transport of the oxidant is the limiting factor to continue the reaction, being insufficient to supply the number of electrons arriving at the cathode.^27,32^ This effect suggests that the Pt/C/CP anode can deliver more electrons to the cathode than the electrode can take by the CO_2_RR at this current density. One final observed feature is the increment in current after 2.5 mA cm^–2^. This feature can be attributed to a side reaction, most likely proton reduction to hydrogen, which occurs due to the high cathodic overpotentials in CO_2_-starved states under mixed media conditions.^33^

The photoexcitation of the CBO/CuO/CP photocathode enhances the performance for all cases. Increasing the flow rate promotes the access of more reactants to the active sites of the catalyst, leading to increased electrochemical conversion. The limiting current density increases from 1.28, 1.93, 2.20, and 2.60 mA cm^–2^ for 30, 50, 75, and 100 μL min^–1^, respectively. This value is collected considering the current density to be equivalent to the sudden cell voltage drop due to starvation (Figure 5). However, the increase in current density with flow rate is not traduced in power density. The maximum power densities are 0.61, 0.74, 0.90, and 0.76 mW cm^–2^ for 30, 50, 75, and 100 μL min^–1^, respectively. Note that there is a maximum at 75 μL min^–1^ and a decrease at 100 μL min^–1^. This suggests the decreased residence time at the highest flow rate may limit the overall conversion.

During the literature review conducted up to this point, we have not found reports of μPFC with CO_2_ as an oxidant. Matsuda et al.^34^ reported a polymer electrolyte fuel cell driven by feeding H_2_ and CO_2_ to the anode (Pt/C) and cathode (Pt_0.8_Ru_0.2_/C), respectively. The cell generated electric power 0.14 mW cm^–2^.^34^ Liu et al.^35^ also reported the CO_2_ reduction to CH_4_ in a conventional fuel cell equipped with a RuO_2_/CNT cathode and a Pt/C anode. The cell comprises a membrane electrode assembly (MEA) and is powered by H_2_ as fuel and CO_2_ as oxidant.^35^ According to the authors, the cell can generate a current density of 94.1 A m^–2^ and a peak power density of 3.9 W m^–2^.^35^

The dark μFC shows positive OCV, suggesting that a small cathodic transient found in the half-cell measurement (Figure 2B) is related to the CO_2_RR. However, the current is too low, making the power densities not relevant (Figure 5). The OCV increases from 0.43 to ∼0.56 V in the presence of light. The OCV of 0.56 V is smaller than that predicted by half-cell measurements (Figure 2D), mainly due to additional polarizations.

Feeding the cell at 30 μL min^–1^ is barely reproducible, as shown by the large error bars in Figure 5A. However, it shows a clear profile, different from the results of the μPFC equipped with a CBO/CP photocathode (Figure S9). At first glance, we observed bubbles inside the colaminar channel at 30 μL min^–1^, which could be a reason for the instabilities. Therefore, we investigated whether the problem of reproducibility for the cells with CBO/CP could be similar.

**Figure 6:**
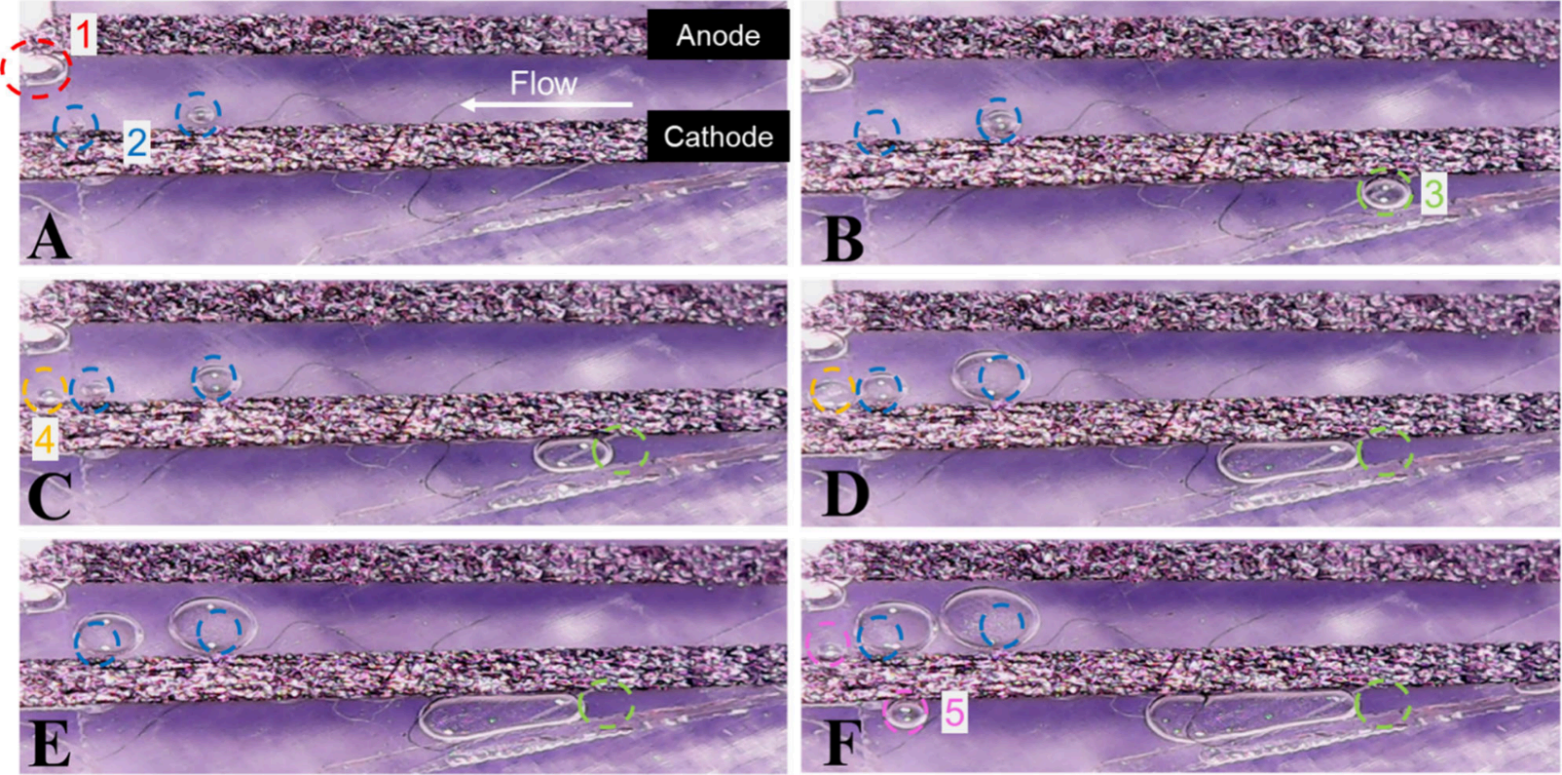
Pictures of the μPFC microchannel equipped with a CBO photocathode fed by 1 mol L^–1^ glycerol + 1 mol L^–1^ KOH anolyte (pH 7, PBS solution CO_2_-saturated catholyte at 75 μL min^–1^, under light of a solar simulator at 200 mW cm^–2^) at (a) 1 s, (b) 15, (c) 35, (d) 80, (e) 100, and (f) 140 s. In all images, the anode is in the upper and the cathode below, and flow is directed from right to the left, as indicated in figure (a). There are bubbles highlighted and numbered in the figure.

We fixed a digital microscope pointed to the bottom of the μPFC equipped with a CBO/CP cathode under light exposure to collect images at an OCV fed at 75 μL min^–1^ of the anolyte and catholyte, as shown in Figure 6. At the instant the cell was exposed to light, we could see bubbles at the colaminar channel (Figure 6A). Although the structures formed within the colaminar channel are cavities,^36^ they will be referred to as bubbles, as suggested by the literature. The red-circled bubble near the anode was referred to as *bubble 1*, and two other bubbles very close to the cathode were circled in blue and named *bubbles 2* (Figure 6A). After 15 s, *bubble 1* remained stable, while group *bubbles 2* increased in size (Figure 6B). At this point, one more bubble appeared near the cathode outside the colaminar channel, indicated as *bubble 3* in Figure 6B. Since the system is operating at the OCV under light, the increase in the size of *bubbles 2* and the appearance of *bubble 3* near the CBO/CP photocathode suggest water reduction into H_2_—photocatalytic water splitting.

The water splitting via pure photocatalysis is more evident with time. After 35 s, *bubbles 2* increased and *bubble 3* increased and moved dragged by the flow, while a new *bubble 4* appeared close to the photocathode (Figure 6C). After 80 s, *bubbles 2* grew bigger than the region first used to indicate them (blue circles), *bubble 3* hugely increased and moved outside its original position, *bubble 4* increased, and *bubble 1* stayed at the same position from the beginning (Figure 6D). Here, we can point out that *bubble 1* could be a bubble of air since the start; moreover, the dark Pt/C/CP anode has no contribution to gas formation or instability of the colaminar channel. After 100 s, *bubbles 2* grew even more as well as *bubble 3*, and *bubble 4* detached from the cathode (Figure 6F). After 140 s, all bubbles kept increasing and moving, and a new *bubble 5* grew at the photocathode.

CBO-based catalysts are efficient for electrochemical water splitting.^37,38^ Therefore, the instability found for the μPFC assembled with CBO/CP is a result of the disruption of the colaminar channel caused by H_2_ bubbles photocatalytically produced at the cathode. Moreover, H_2_ bubbles may be oxidized at the cathode due to the high experienced potential, competing with the CO_2_ reduction reaction. It is well-known that the presence of bubbles at electrode interfaces, whether they are the desired product, secondary products, or products of competing reactions, affects the efficiency of electrochemical processes.^17,39−42^ However, in the case of microfluidic cells, whose principle of operation is based on the stability of laminar flow, this phenomenon limits the functioning of the device, as it completely disrupts the laminar flow channel.

Decorating CBO with CuO to create the CBO/CuO heterojunction results in a Fermi-level alignment in such a way that *E*_*V*_ and *E*_*C*_ of CBO move negatively in the SHE scale and those from CuO move positively as shown in Figure 3. It induces electron transfer from CBO to CuO, with *E_c_* at energy more positive than −041 V vs SHE, limiting photocatalytic water splitting. A thorough discussion of the energy band alignment for this heterojunction can be found in our previous publication.^18^ Therefore, adding CuO to CBO is a strategy to surpass water splitting at the cathode, allowing the use of μPFC to convert CO_2_ into power by using an aqueous solution.

It is important to note that the use of CBO/CuO prevents HER but does not avoid it completely (Figure S4). Thus, the cathode may face HER as a side reaction to some extent but, obviously, in a very small amount compared to the cell equipped with CBO cathode. Moreover, the presence of nondissolved CO_2_ gases also may disturb the colaminar channel. The potentiodynamic measurements reveal a stable system for potential transients. The long-term potentiostatic measurement shows that the μPFC equipped with CBO/CuO/CP photocathode operates for ∼110 min, as shown in Figure S10. Operating at 0.45 V of cell voltage, the μPFC delivers ∼1.9 mA, leading to ∼0.86 mW cm^–2^ at 75 μL min^–1^ (Figure S10). The performance decrease with time up to 1.8 mA after 110 min.

In summary, we have shown the first μPFC for the conversion of CO_2_ into power in an aqueous solution. For that, we have first demonstrated how to seal the cell with a glass transparent to visible light and partially transparent to UV. This strategy consists of using the adhesive from touchscreen protective films to seal the cell. The photocathode also promotes water splitting, so we used a CBO/CuO heterojunction as a photocathode to avoid most of the H_2_ evolution and enhance the CO_2_RR. The spontaneous galvanic cell converts CO_2_ and glycerol into 0.9 mW cm^–2^ as the maximum power density. This unprecedented work shows that new engineering strategies allied with the use of new semiconductor design help develop devices to capture and convert CO_2_ with zero bias.

## 4 Conclusions

This work presents a significant advancement in the microfluidic fuel cells (μPFCs) field, particularly in their application for the conversion of CO_2_ into energy. Our development of a hybrid μPFC, integrating 3D-printed components with glass sealing, represents a significant engineering advancement. This novel design not only ensures operational stability but also allows for the efficient utilization of light-transparent glass, enabling the exposure of photocatalytic components to radiation necessary for energy conversion.

By conducting half-cell measurements and cell tests, we have provided insights into the electrochemical behavior and performance of our μPFC under various conditions. Fundamental half-cell measurements revealed that it is possible to couple glycerol electrooxidation on Pt/C in an alkaline medium with the CO_2_ reduction CBO or CBO/CuO at neutral pH in a spontaneous overall reaction exposed to solar light, enabling the construction of a galvanic cell. Both semiconductors present similar current densities for the CO_2_RR; however, CBO/CP presents a higher current for the HER since the electrons in the CBO conduction band have enough energy to conduct H_2_ generation. Therefore, decorating CBO with CuO rises as a strategy to reduce the electron reduction ability by making a type-II CBO/CuO heterojunction that avoids water splitting, leading to more efficient CO_2_ reduction. This heterojunction exhibited a stable and reproducible performance, surpassing the challenges associated with photocatalytic water splitting and enabling the efficient conversion of CO_2_ into power.

By combining insights from materials science, photo(electro)chemistry, and engineering, we developed a cutting-edge μPFC capable of converting CO_2_ into energy (0.9 mW cm^–2^). This represents a significant step forward in the quest for sustainable energy solutions and offers promising avenues for future research and development in this field.

## Supporting Information Available

Details of materials synthesis. Details of the cells and experiments. Calculations of Reynolds number. Polarization and power density curves (PDF)
