# Supplementary material for: Converting Energy with Glycerol and CO2 in a Microfluidic Fuel Cell Equipped with CuBiO4/CuO Photocathode: Bypassing Bubbles Challenge of Concurrent Water Splitting
Source: ACS Omega. 2024 Oct 15;9(43):43658–67. doi: 10.1021/acsomega.4c05943 (PMC11525488; doi:10.1021/acsomega.4c05943)
Supplement: Supplementary file 1 — ao4c05943_si_001.pdf [file ao4c05943_si_001.pdf]

Supporting Information

Converting energy with glycerol and CO_2 in a microfluidic fuel cell equipped with $CuBiO_4$/CuOphotocathode: Bypassing bubbles challenge of concurrent water splitting

Silvio M. Mazarin,[¥] Daniel F. Costa-Filho,[¥] Cinthia R. Zanata,* Adailton C. Nogueira, Maria-Victória S. Silva, Heberton Wender, and Cauê A. Martins[a**]

Institute of Physics, Universidade Federal de Mato Grosso do Sul, CP 549, 79070-900, Campo Grande, MS, Brazil.

*cinthiazanata@gmail.com

**caue.martins@ufms.br

* Corresponding Author. Phone: +55 67 99262 4202

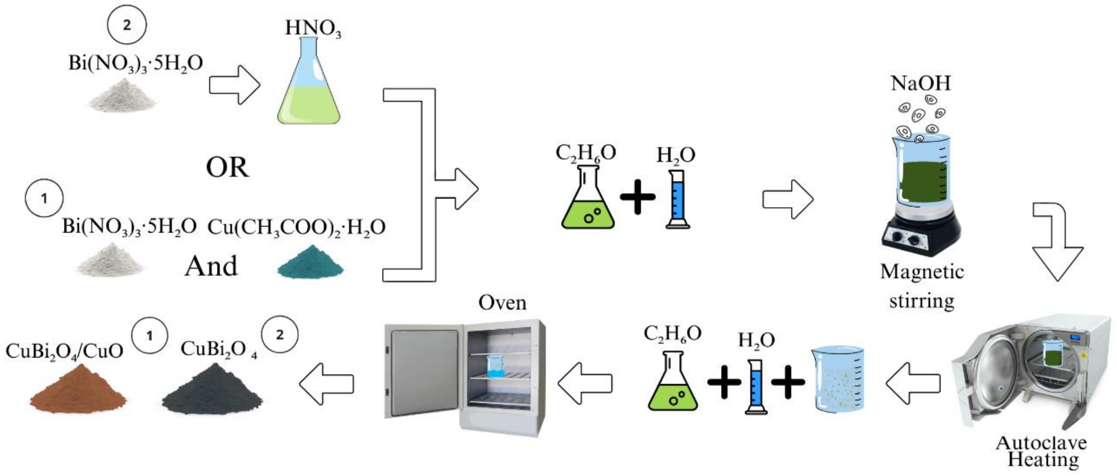

Figure S1. Schematic of the synthesis process of the photocatalysts $CuBi_2O_4$ (CBO) and $CuBi_2O_4/CuO$ (CBO/CuO) using the solvothermal method. Route (1) yields CBO/CuO and (2), CBO. Method based on previous works.[1]

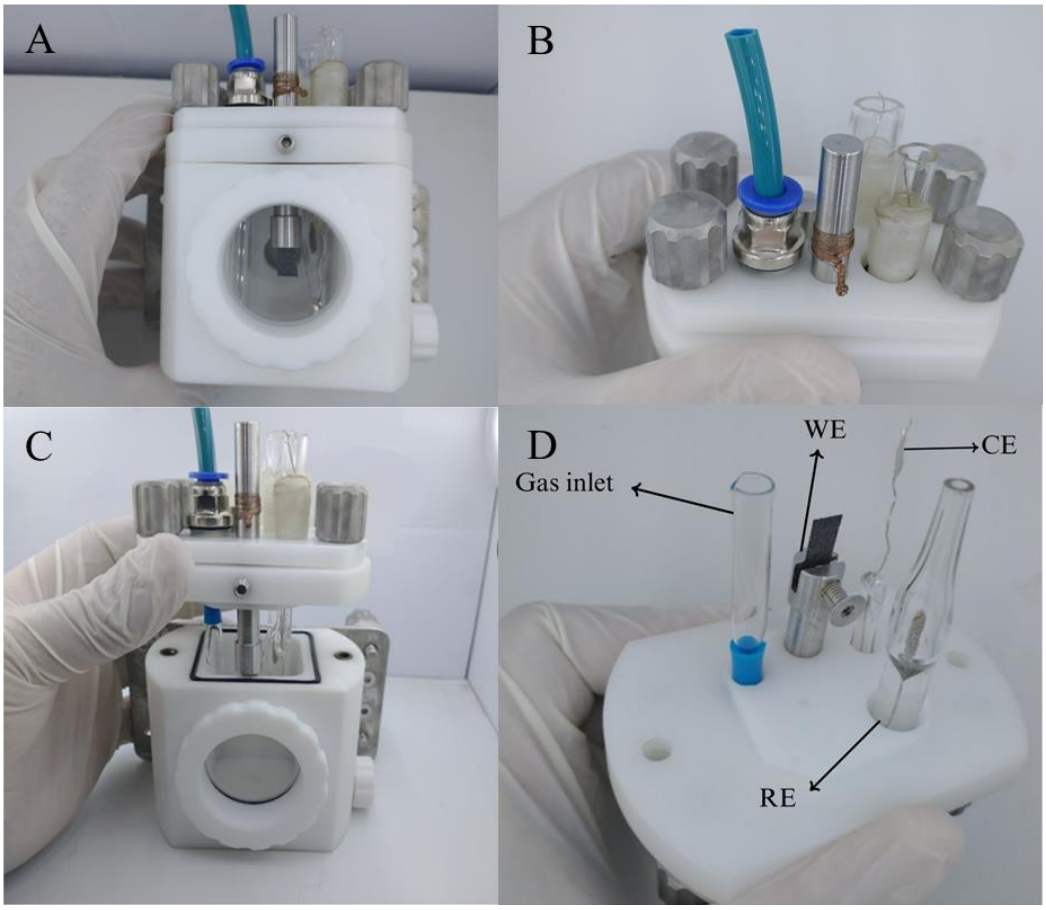

Figure S2. Details of the reactor for half-cell measurements with light incidence, featuring (a) a general view of the assembled cell, (b) a Top view of the open lid, (c) a front view of the disassembled cell, and (d) details of the electrodes and gas inlet.

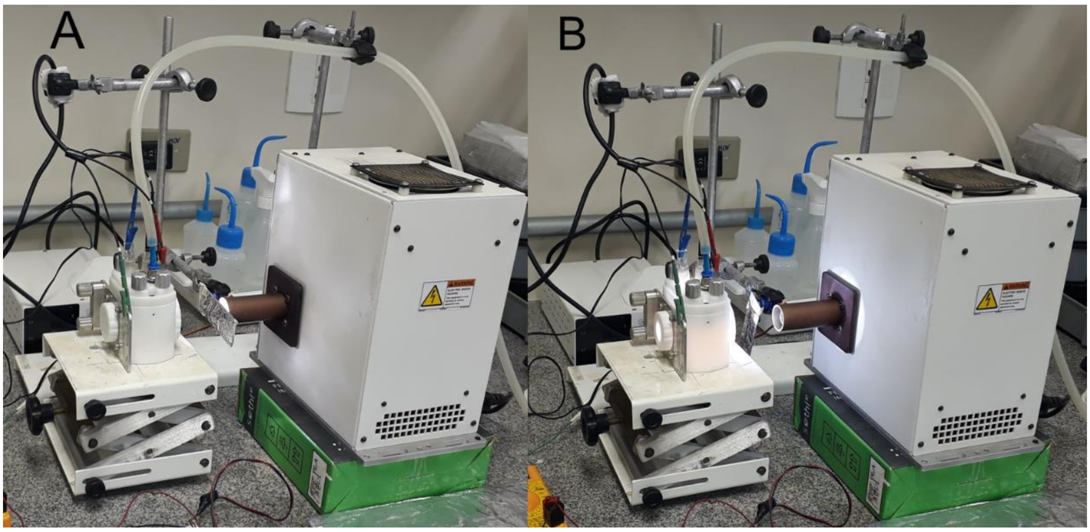

Figure S3. Operation of the light incidence control device for half-cell measurements (a) under dark and (b) under light.

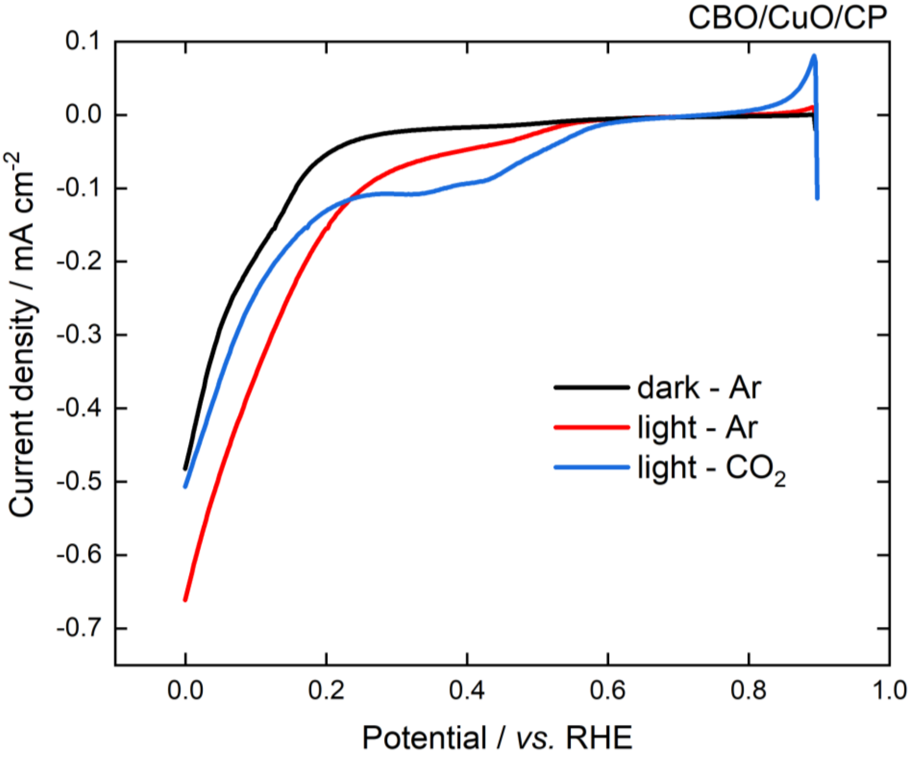

Figure S4. Electrochemical measurements in half-cell measurements, featuring linear voltammogram of CBO/CuO/CP in the presence and absence of CO_2 in PBS (pH 7) at dark and under dark and light conditions. All measurements performed at 0.01 V s^{-1}. Measurements under light were performed at 200 mW cm^{-2} light intensity. The potentials of the reversible hydrogen electrode (RHE) prepared in the electrolyte (same solution) as a reference were corrected to the standard potential of the reference electrode, calculated using the Nernst equation $E = E^o + 0.0591$ pH.

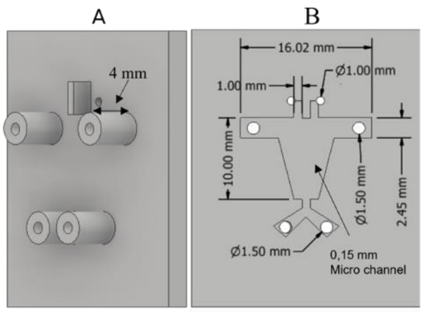

Figure S5. Details of the 3D-printed microchanel.

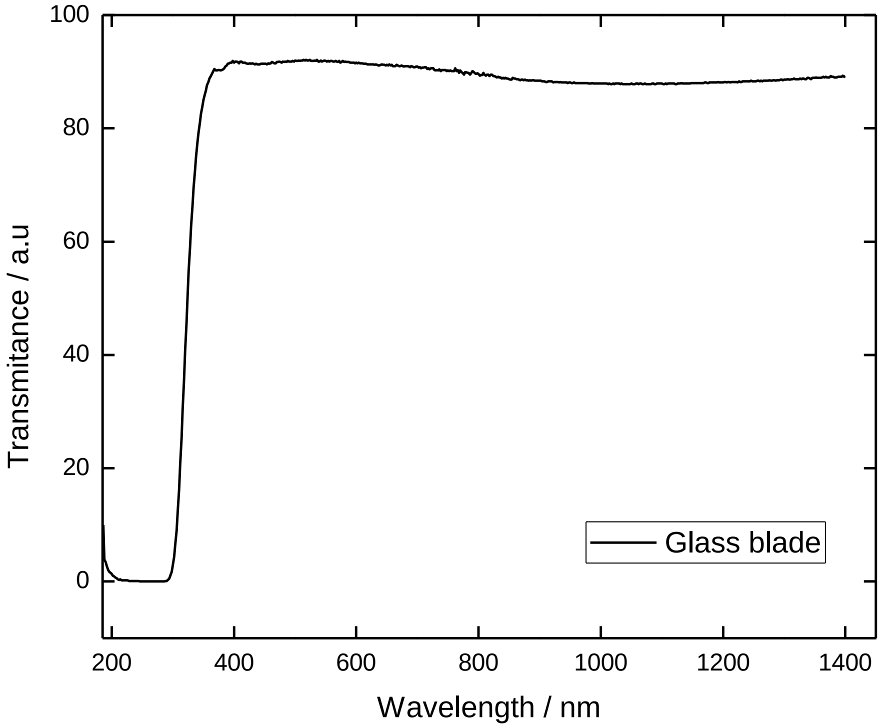

Figure S6. Spectral transmittance of the glass used to close the microfluidic fuel cell.

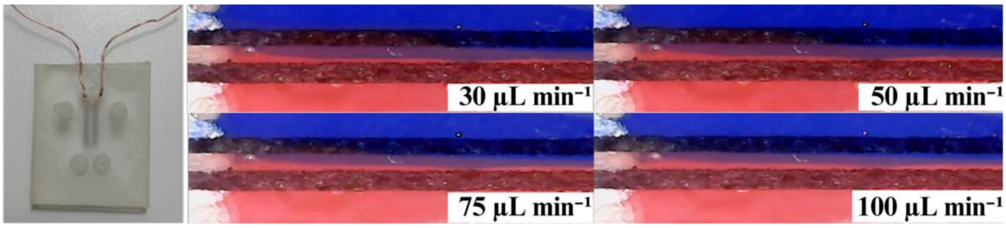

Figure S7. Pictures of the µPFC and of the glass bottom displaying the flow of inks at different flow rates.

Calculation of Reynolds' number based on fluid density ($\rho = 997\ kg\ m^{-3}$), average velocity ($U = Q/A$), hydraulic diameter (D_h) and dynamic viscosity ($\mu = 8.9\ 10^{-4}\ kg\ m^{-1}\ s^{-1}$), where A is the cross section of the microchannel, given by the product of its width (w) by its height (h).

$$Q = 30\mu L\ min^{-1} = 0.5\ 10^{-9}\ m^3\ s^{-1} \qquad Q = 75\mu L\ min^{-1} = 1.25\ 10^{-9}\ m^3\ s^{-1}$$
$$Q = 50\mu L\ min^{-1} = 0.83\ 10^{-9}\ m^3\ s^{-1} \qquad Q = 100\mu L\ min^{-1} = 1.66\ 10^{-9}\ m^3\ s^{-1}$$

$$A = w \times h = 0.003 \times 0.0015 = 4.5\ 10^{-7}\ m^2 \qquad D_h = \frac{2(b \times h)}{w + h} = \frac{2 \times 0.003 \times 0.0015}{(0.003 + 0.0015)} = 2.86\ 10^{-4}\ m$$

$$U_{30} = \frac{Q}{A} = \frac{0.5\ 10^{-9}}{4.5\ 10^{-7}} \rightarrow U_{30} = 1.11\ 10^{-3}\ m\ s^{-1} \qquad U_{75} = \frac{Q}{A} = \frac{1.25\ 10^{-9}}{4.5\ 10^{-7}} \rightarrow U_{75} = 2.77\ 10^{-3}\ m\ s^{-1}$$
$$U_{50} = \frac{Q}{A} = \frac{0.83\ 10^{-9}}{4.5\ 10^{-7}} \rightarrow U_{50} = 1.84\ 10^{-3}\ m\ s^{-1} \qquad U_{100} = \frac{Q}{A} = \frac{1.66\ 10^{-9}}{4.5\ 10^{-7}} \rightarrow U_{100} = 3.55\ 10^{-3}\ m\ s^{-1}$$

$$Re = \frac{\rho U D_h}{\mu}$$

$$Re_{30} = \frac{997 \times 1.11\ 10^{-3} \times 2.86\ 10^{-4}}{8.9\ 10^{-4}} \rightarrow Re_{30} = 0.36 \qquad Re_{75} = \frac{997 \times 2.77\ 10^{-3} \times 2.86\ 10^{-4}}{8.9\ 10^{-4}} \rightarrow Re_{75} = 0.89$$
$$Re_{50} = \frac{997 \times 1.84\ 10^{-3} \times 2.86\ 10^{-4}}{8.9\ 10^{-4}} \rightarrow Re_{50} = 0.59 \qquad Re_{100} = \frac{997 \times 3.55\ 10^{-3} \times 2.86\ 10^{-4}}{8.9\ 10^{-4}} \rightarrow Re_{100} = 1.14$$

Figure S8. Calculation of Reynolds's number (*Re*) for 50 and 100 µL min^{-1} based on fluid density (ρ), average velocity (U), hydraulic diameter (Dh), and dynamic viscosity.

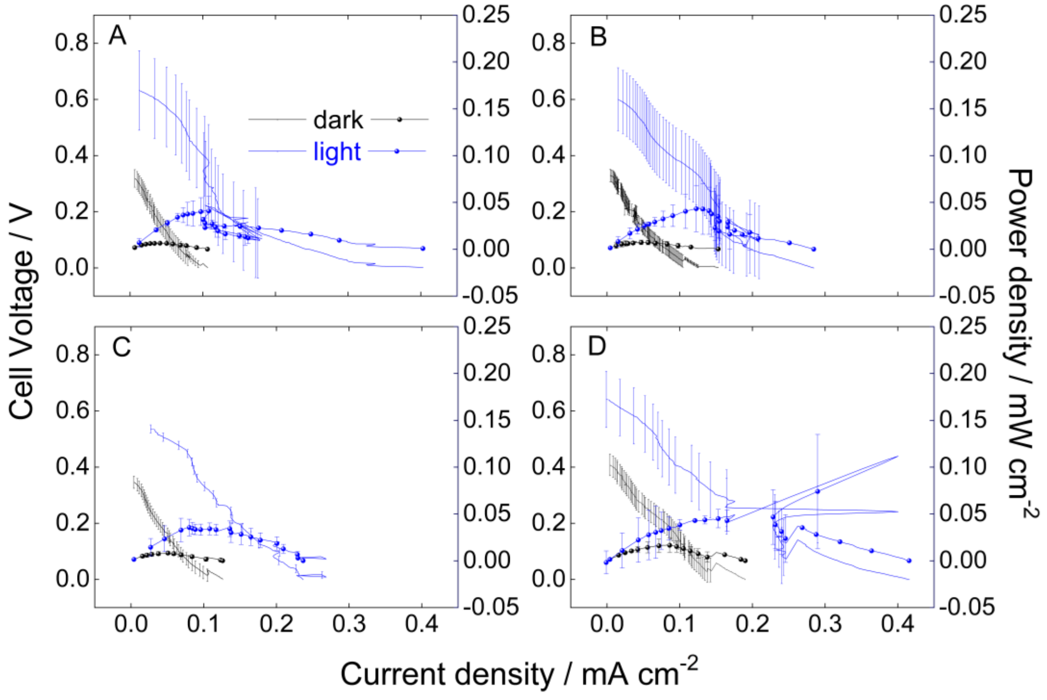

Figure S9. Polarization and power density curves of a µPFC equipped with CBO/CP photocathode and Pt/C/CP dark anode fed by 1 mol L^{-1} glycerol in 1 mol L^{-1} KOH anolyte and CO_2-saturated PBS catholyte at (a) 30, (b) 50, (c) 75, and (d) 100 µL min^{-1}. Measurements were collected from OCV to 0.01 mV at 0.01 mV s^{-1} under light induced by a solar simulator at 200 mW cm^{-2}.

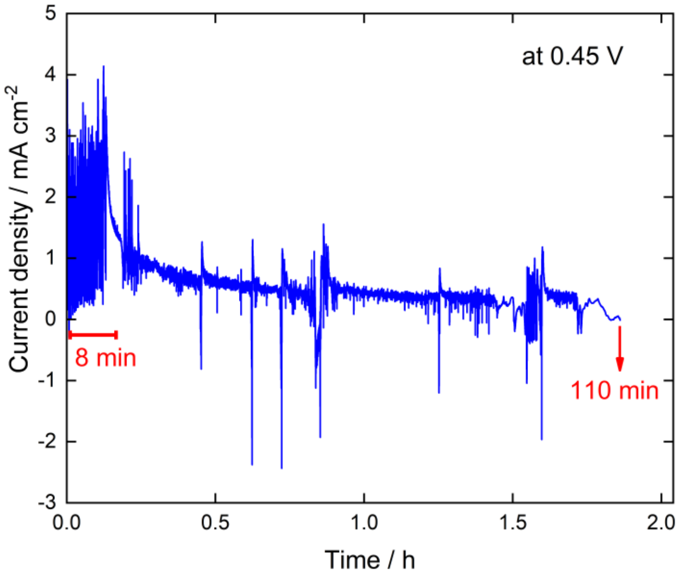

Figure S10. Potentiostatic long-term measurement of a µPFC equipped with CBO/CuO/CP photocathode and Pt/C/CP dark anode fed by 1 mol L^{-1} glycerol + 1 mol L^{-1} KOH anolyte and CO_2-saturated PBS catholyte at 0.45 V of cell voltage at 75 µL min^{-1}. Measurement were collected under light induced by a solar simulator at 200 mW cm^{-2}.

REFERENCES

1 A. C. Nogueira, L. E. Gomes, J. A. P. Ferencz, J. E. F. S. Rodrigues, R. V. Gonçalves and H. Wender, *J. Phys. Chem. C*, 2019, **123**, 25680–25690.
